# Human Values in Medicine
*Delivered to the Bristol Medico-Chirurgical Society on 8th May 1974.


**Published:** 1976

**Authors:** 


### Human Values in Medicine*

**Metropolitan**
**Anthony of Sourozh**

Our President has been wise enough to give all his appreciation of the lecture I am going to give before he can hear it, so that he can do it in all sincerity, simply on the grounds of hope.

I am afraid I have really little right and reason to be here speaking on my subject, yet Dostoyevsky said in one of his writings that if you are really concerned with something, if you earnestly are interested and committed to something, you have both a right and a duty to speak on it. I am not going to unveil before you what Dr. Barbour called the "tricks of the trade", I am not going either to attempt at discussing the values, ethical problems, which confront us nowadays in detail. I would like to speak of some basic facts of the human values in relation to medicine and dwell perhaps a little bit more on the question of suffering in general and the question of death, its place within our medical, Christian, priestly situation, because I happen to be simultaneously a physician that has defected and a priest.

Immediately after the war, in conjunction with the Nuremberg trials and the various investigations that were made concerning concentration camps, documents came out about the way in which prisoners had been used as guinea pigs for medical research. This is a point at which I would like to start, not indeed to discuss what happened or to describe it but to underline the fact that, from the point of view of the medical tradition, from the point of view of the tradition of medicine since the earliest times, a patient can never be considered as or treated as an objective investigation, as a guinea pig. What makes medicine, I think, a very peculiar branch of human activity is the blending in it of science and of values, approaches, which have nothing to do with science. Compassion is at the root of the medical concern and compassion is not a scientific approach by its very nature. It is a human approach, it can be introduced into, applied to, any branch of human activity, but apart from compassion, without compassion, there is no medicine. A physician who has been nothing but a man of science capable coldly, coldbloodedly, dispassionately to do the right thing without relating to a patient at all, without having any interest in the patient but only in the exercise of medicine, either in drug therapy or in surgery or in other methods, would not be a physician in the sense in which I hope, I wish we all think of medicine.

I remember a young surgeon who has now a chair of surgery in France with whom we were discussing before the war the advantages or otherwise of anaesthetics in one or another operation, and he said plainly that the only purpose of an anaesthetic was to make the work easier for the surgeon. Whether the patient suffered or not was totally irrelevant, and I am not putting words into his mouth, it was exactly what he said and meant, he would have carved a patient live if that could be done without disturbance and could be done without making his operating more difficult and more unpleasant—to him. I have met during the war also a young surgeon, not of the French army, who was a prisoner of war. He had a right of access to the soldiers and officers of his own nationality. I offered to serve for him as anaesthetist. He shrugged his shoulders and said, "It's soldiers we deal with, they must be prepared to face suffering", and he operated on them without anaesthetics whenever it created no problem, and I remember again in one of the cases of his operations a soldier who had an enormous abscess of the leg whom he refused to use anaesthetics for. He operated just like this, the man howled and cursed, and when he had finished and the man had recovered some of his poise, being a well disciplined and well trained soldier he apologised to the lieutenant for the language he had used, and I remember the lieutenant saying to him, "That's all right, your language was just in equipoise with your pain, I forgive you," but it never occurred to him that the pain was in equipoise with his inhumanity and his complete lack of sense of solidarity.

I am giving you these examples because, although they do not occur every day, there are situations in which people get hardened and there are people who in the most normal situation can be so cold and so totally devoid of human compassion, of perceptiveness, that they lose the right to be thought of as physicians. They are butchers, they are technicians, but not physicians. The French writer La Rochefoucauld says in one of his writings, "One has always enough courage to bear other people's sufferings." This is exactly what a physician should not be and should not do. At the root of the medical attitude to the patient, to the problem of illness, to all the ethics and philosophy of medicine here is this virtue of compassion, this sense of solidarity, this respect and veneration for human

*Delivered to the Bristol Medico-Chirurgical Society on 8th May 1974.*

life, this devotion to the only person who is now in his presence. Short of this the medical profession may be remarkably scientific but it would lose its very substance.

Now, compassion does not mean sentimentality. Those of you or those of us who have any experience of tragic situations, of surgery or of emergency medicine, particularly under circumstances and situations of strain, know perfectly well that we must remain unemotional, at least in the process of treating a patient. You cannot operate under fire in a state of emotion, but all your concern must be on the patient whether people shoot at you or not, because he matters more than you, you are there for him, you have no other raison d'etre than him, his need. Compassion is not the kind of empathy which we may feel at times, which at times is easy to perceive and at times is induced at the cost of great imaginative efforts. It is not an attempt at feeling what the other feels. This is simply impossible, no-one can feel one's neighbour's toothache, not to speak of more complex emotions that go together with the discovery that you have cancer or leukaemia, that death is there waiting for you, looming over you. I remember one of my parishioners who lost a child and a young curate coming up to her after a service and saying how sorry he felt, and he added, ill advisedly, "I understand you so well," and the good lady turned, rounded on him and said, "Fool, you understand nothing. First you are not a mother, secondly you have never lost a child, you cannot understand what I feel."

What you could do is to feel pain, your own pain, on occasion of my suffering. Well, this is a very important distinction because we should undergo an education, accept an education, and educate ourselves in the ability to respond with all our understanding, all our heart, all our imagination, to what happens to others, but not try to perceive almost in a visceral way, in a biological way, a pain which is not ours, an emotion which is not ours. Our patient is in no need of our perceiving his pain or his suffering, what he needs is our creative response to his suffering and his situation, and a response that is creative enough to force us into an action, an action which is rooted first of all in the respect, in reverence for this person, not for an anonymous patient, not for No. 7 in Ward 13, but for a person who has got a name, a surname, an age, features, who has got a husband or wife or a fiance or a child, someone who must become to us concrete to the utmost and whose life consequently matters, not simply because we have a general attitude to life and we were taught that we are there to preserve life, to make it last as long as possible and so forth, but because this particular person, whether I like this person or not, matters. To whom?

That we can answer in different ways according to our faith or lack of faith. If we are Christian, if we believe in God altogether, if we believe that no-one comes into this world otherwise than called into it, willed into it, loved into it by a God of love, then this person matters at least to God, but also, and we forget that very easily, I am afraid, there is no-one that does not matter to someone. That applies particularly to people for instance in prison or to war criminals or to people whom we charge with inhumanity. Whoever it is has a mother to whom he matters, a wife, a brother, a sister, and so forth. Perhaps the closest the people who truly love him or her whom we feel one should not love but only condemn, know only one side of this personality. It may well be that the side which they know is as real as the one which we know and which they do not know. I am reading at present Solzhenitsyn's *Gulag Archipelago.* In one of the chapters he underlines this fact, that a man, a person, man or woman, is seen in so many ways by the various people who come into contact with him, and he quotes a conversation that was reported to him. One of the hardest, most heartless interrogators was married and one day his wife, who knew him from a quite different angle, said to a friend, "My husband is such a good interrogator, he told me that there was a man who for weeks and weeks would not recognise his crimes and then he was sent to him and one night's conversation made him avow everything." This woman had no idea of the way in which this recognition of his wrongs had been obtained. She did not know this man from this angle, and this of course is an extreme example, but none of us knows other people from all angles.

How little we know ourselves, how little we suspect what kind of person we can become, may become, under unexpected circumstances, not under duress but simply because we are of a sudden no longer a person known but a person that has become anonymous. So many things happened during the war to people, are done by people, because of their anonymity, because he has no name, he is just one of the many soldiers. Now, I am insisting on this point because it is very easy in certain situations to say that the life of this person matters nothing while the life of another person does matter. Leaving aside the absolute value which God attaches to each of us, the fact that if we ask ourselves or the scriptures, what is the value of any of us in the eyes of the Christian God, we can answer, all the life, all the death of Christ. But also, as I have already said, no-one, not one person, is alone in the world, there is always someone to whom he matters and our attitude as physicians should be a devotion to life, not simply in general terms but in the concreteness of this recognition, he matters, she matters, however incomprehensible it be to me, there is someone for whom his death, his suffering, is acute pain and real tragedy.

There is also in the relationship between physician and patient another side which is connected with this sense of compassion, this attitude of human solidarity, this reverence for his personal, unique unrepeatable existence. It is the way in which a patient surrenders into the hands of a physician and trusts himself to a physician. Now at this point there is an element that comes into the picture which is to me very important.

The physician is a man or a woman who is aware of the significance and I would say the sacredness of a human body. As long as we are well we think of ourselves as though we were spiritual beings. We have got of course a body that allows us to transport ourselves from one place to another, to act, to enjoy life, the five senses with which we are endowed, the mind, the perceptiveness, all that we see in terms of our spiritual being. We take our body for granted, in a way we try to enjoy it as much as we can but we do not, or so seldom, no, we do not simply think of our body as though it was a partner on equal terms with our soul and yet when this body decays, when illness, pain, hits our body, then of a sudden we discover that I am my body. I am not my distressed mind, my dismayed emotions, I am this body which is now in danger of destruction, which is full of pain, and the pain which we endure does not need to be the pains of cancer, it may be toothache that can drive you up the wall. I tried once to be ascetical and heroic, I had a toothache and I thought, I will just stand it, am I not pure spirit? My pure spirit endured it during the day with increasing difficulty, then the night came and I couldn't sleep, strangely enough my soaring spirit was kept awake by this miserable body of mine, and about two o'clock in the morning I went to the tool cupboard of the place where I was and I got my tooth out with the tongs which were used for nails.

Now, I became aware at that moment that my body and me in a very strange way could be identified, they were extraordinarily close to one another. Now, when someone is truly ill doesn't he, everyone, each of you, don't we all discover how much our body matters? What if this body dissolves, what if it becomes an unseemly, distasteful, rotting mass of flesh and bone? Well, think of leprosy, think of so many other diseases that can transform our body into a repulsive, revolting thing. And then think of the way in which you bring yourself, your body, indeed your proud and beautiful spirit brings this body to the physician and says, here am I, helpless, hopeless, afraid, I am ill, I don't know what to do but you can save me. Help, be kind to this body, treat this body with reverence, treat this body with care, and how grateful we are when the physician to whom we have come treats it with reverence, with chastity. How grateful we are when we discover that the physician to whom we have entrusted this body understands what a human body is, not simply material support for our great spirit, but us, us to such a degree that, if that body goes, where am I?

All this is a background of the human attitude of the physician. Now, there are innumerable problems that are connected obviously with medical ethics. I would like to concentrate on two things. One of the elements of the Hippocratic oath, or of the ancient oaths of the doctor, was that he would preserve life and alleviate pain. There was no problem about the scale and scope of his activities up practically to the last war. It is only after the last war that things got out of hand in the sense the we are now in possession of drugs or surgical techniques or other techniques that allow us to kill pain to a degree unheard of before, to kill psychological anguish and pain and to prolong life. Now, to what exent can we follow this line? Is it legitimate, and possible, to go ahead endlessly along this line, or are there criteria which will allow us, or force us, to enter into a partnership with pain and with death? I will explain this word of partnership by one example. Some of you may have read a book which was very popular in the thirties, Axel Munthe's *Story of San Michele*. I read it in those days and I can't remember the detail of what I want to put to you, but the substance is this. When he was a young medical student in the Hôtel Dieu of Paris, where I started my medical training, to begin with he had an impression that the whole of medicine was a fight between the doctor and the enemy that was death. Death was to be defeated, death was to be hated, death could not be accepted, everything was to be done against death, and then, observing all the physicians and perhaps more particularly the nurses, he discovered that there was a much more subtle relationship between the physician and death over the patient. There was a moment when one could fight for life, hopes were high, medical possibilities offered more or less hopes, and indeed at times these hopes were rewarded, but then in other cases with other patients, in spite of all that had been done life could not resist the onslaught of decay, of illness, whether it was infection or cancer or TB or age, and then he noticed with surprise, and then with increasing interest, with a sense that grew deeper and deeper in him, that a new partnership was established between death and the physician, and there was a moment when things were as though the physician turned to death and said, my time is over, yours has come, let us work together, take over and be kind.

I think this attitude to death is very important. It corresponds to simply the realities of life. Whether we are believers or atheists, we are confronted with the same fact, that there will be a moment when struggling, fighting over this human being to prevent him from dying will transform this body and this mind and this heart into a battle field that will be torn, that will be tramped upon. It will no longer be a struggle for this person, it will be an anonymous struggle, an anonymous battle against death irrespective of what this person endures in the process of this fighting for the preservation of his life. Now, there are in my experience, and I must say, perhaps sadly, that my experience of dying people is extensive, within my family and outside of my family, in the years of war, in the years of hospital training and of practice as well as in the 25 years that I have been a priest, there are two kinds of people who face death with peace. They are rare, comparatively. It is the people who truly believe and those people who truly do not believe. The people who cannot face death are the people who are half believers, or quarters of believers, underbaked, people who do not believe in life, in eternity, in God, but who at the same time are not sure that annihilation is real annihilation, or if one could think in terms of a true annihilation, of not being there, there would be in a way no problem, or less of it, but where things go wrong, where things are very difficult—and you know how illogical our mental processes are—so many people think, yes, but how horrible it will be to discover that I am no more—oh, you laugh, ask yourself how logical you are in other walks of life and how sure you are that you yourself, when you think of your death, not someone else's death but your own, do not feel that it will be horrible to fall into nought and to see that I am an empty space, there is nothing left of me.

Of course from any logical point of view it's absurd, but absurd we are most of our life. People who do not believe, who truly are certain of annihilation, can die. I have seen it, and also people who are faced with death at a moment and in a way which makes sense of their death. I am not extolling the example which I will give you as something which is right as far as its substance is concerned, but as far as the attitude is concerned. During the battles of June 1940 I was on the front line receiving wounded people and I was told that in a corner of our tent there were two dying Germans. As I speak German I was asked to say a few words to them for them to die in less loneliness. They were so appallingly destroyed by heavy machine gun that it was really beyond description. I turned to one of them and, simply to say something, I said, "Do you suffer unbearably," and he smiled and said, "I don't suffer, we are beating you". I don't mean to say that his reason for forgetting his suffering was right in itself, but this is the kind of thing which the martyrs would have said, which a mother could say when the life of her child is involved.

If we have a reason that makes sense of our death we can face it, when we have a faith that allows us to see death as one of the stages of life we can face it, not otherwise, but where the physician comes into the picture, and where there is a dilemma for the physician, is this. In the present situation we are not, or rather you are not asking a patient what he thinks of his life and of his death. You force him into living, you force him rather not into living, into existing, into enduring, in both senses of the word, both lasting and outlasting himself and enduring all the heaviness and pain and anguish of this lasting longer than he wants to, and here there is an ethical problem for the medical profession, but how can this problem be solved? Not otherwise than by a consideration of human values and by non-medical factors because, unless we have a definite attitude to life and its values and to death and its place and significance, we have no other alternative than force people to live until they escape us in the end with a sigh of relief, out of our hands and enter into peace, but this is a problem which must be faced by the medical profession, must be an object of reflection by the medical student. Yes, life is the ultimate value, but is simply lasting identical to living? Yes, death for a christian is the last enemy to be defeated, but is it defeating death to force someone to remain artificially alive when no life is left. Again it is true that forcing a life to continue is not part of our human struggle for the victory of life over death? I am not solving the problem for you, I am setting it for you. I have my own feeling about it.

Suffering comes also into the picture. We have now unheard of possibilities to alleviate physical suffering and moral anguish and agony. Is it right to use these means? At first you will probably simply shrug your shoulders and say, "Of course it is, aren't we there to achieve it, isn't it what each sufferer expects from us?" Yes, he may, but at what point should we intervene and to what extent? Again it is a question of human values, what is the value of a person, the moral value of a person who, confronted with bereavement, prefers to be made insensitive, prefers to escape the pain and suffering and agony of the bereavement? What does it say about the relationship there was between this person and the person who died? This attitude is conditioned by an attitude to life and an attitude to death and an attitude to self, by fear, by many things, but is it humanly dignified, can one vindicate this attitude? Would any one of us wish his wife, his mother, his daughter, to refuse to face the pain of his death and say, "I want to forget, I want to feel as though he was not dead," or as though it did not matter. What does it say about human relationships, of which we speak world without end and ad nauseum? What does it say about my love to the beloved one when I say, now that he, she, is dead, better forget about it, better become insensitive, because it disturbs me. And again, what is the human value of a person who is so afraid of suffering that he will never, or she will never, face any kind of physical ordeal for fear of the suffering?

I was brought up perhaps in a strange way, I am grateful for it. As far as death is concerned, I remember a phrase of my father's, or rather two phrases. When I was very young we spoke of death and, without explaining to me what it was because it is something which one must discover, he said to me. "Learn in the course of your life to wait for your death, as a young man waits for his bride." And on another occasion, as I was coming back from a holiday camp he met me and said, "I was worried about you." Flippantly, I answered, "Did you think I had broken my neck?" and my father, who loved me but in a sober way, said to me, "No. That would have mattered little. I thought you had perhaps lost your integrity," and he added, "Remember that whether you are dead or alive is of no importance, even to you. What matters is what you live for and what you are prepared to die for." It applies to death, it also applies to suffering. What am I prepared to stand up to? What education shall I receive from my doctor with regard to my suffering? The easy thing is to give generously enough tranquillisers and aspirin and phenobarbitone and whatnot for all your patients to feel no pain. At what cost? For one thing, it is only against odds that our character becomes vigorous, courage grows, the ability to fight and to stand for values grows. Are we there to undermine this, to encourage cowardice, to allow people to live by fear and for fear's sake to run away from the challenge of life, of death, of pain? On the other hand, and this I think can be observed quite easily, isn't it true that when you begin to alleviate pain you begin with standing up to a certain measure of it, then less and then less, and when you are sure that you can be saved from pain at any moment, then a new pain comes into the picture, the fear of pain? There are people who take aspirin in case their teeth ache. Now you may say, "What is it to us physicians, the patient comes and I am there to respond to his need." No, we are not there to respond to his need, not any more than I as a priest am there to respond to a need. We are not a shop, we are not a restaurant, we are not a circus, we are not simply there to deal out what we are commanded to deal out. In the modern society where people have, at least in theory, an increasing sense of community, of mutual responsibility, of solidarity —I am not speaking of higher qualities of love because they are far above what we can usually afford—we have a duty to confront our neighbour, and ourselves first of course, with the challenge of being human, and being human is a great thing, it entails daring, fearlessness, creativeness, and it is not above human capabilities, only we do not use these capabilities enough.

I remember Solzhenitsyn, who is now in the limelight. He was arrested a few years ago and taken by the KGB and had one of those homely conversations that one can have with political police. He was told that he must keep quiet, he must no longer write, he must no longer speak, he must become like everyone, and he said he wouldn't. Then he was told, but do you realise that you have been already in prison, in camp, do you realise all we can do to you, and he said, "Yes, you have done to me all you could and you have not broken me and I am not afraid of you any more." We admire him but can we emulate him if we are not prepared to face up to odds? Do you imagine that being for a number of years in prisons and concentration camps is a sort of charisma that gives a man endless endurance, heroic courage? No, you come into prison, you go into camp with the amount of courage which you possess. Of course, and that is another aspect of the question, if we allow ourselves to live on too low an ebb, if we are content with just crawling along instead of living, of being a bog instead of being a stream, then of course we cannot summon anything out of ourselves. I would like to give you an example, not theological, not medical either. During one of the bombardments of Paris I was on furlough and found myself on the 4th floor of a house with my mother and grandmother. We never went downstairs into a shelter because we always felt that falling from the top would be a nicer thing than being buried underneath. We sat for a while and then my mother made a very enticing suggestion, to go to the kitchen and on a heap of matches which we carefully had collected, to make a little fire, warm a little water and call that tea, which was all we had. She went into the kitchen, then there was a blast and there was a cry, and I thought my mother was wounded. She never was afraid of bombardments, she had had a daring life before the revolution, so I rushed into the kitchen and I found my mother on all fours on the kitchen table pointing to the corner of the kitchen and saying, "There, there", and there was a mouse. Now, I wonder whether you have ever realised how difficult it is to look up to a mouse? It is too small, it's down below, you can look up at something great that challenges you but the moment you do this, there is no mouse, the moment you do this, there is no person.

Well, this applies to suffering, this applies to our attitude to death. I do believe, and I would like you to believe also, that the role of the physician in a responsible society that wishes to build a city of men worthy of men and, for those of us who are believers, a city of man which would be coextensive with a city of God, is as a priest, indeed as any member of society, but in his own particular way, has got to take part in the challenge of life, not to be one who makes people soft, cowardly, helpless, but a profession that has a vision of life because we have a vision of death and their equipoise, because we have a vision of what man is and because we are not prepared to allow either ourselves or anyone else to be below human stature.

I have spoken much longer than I promised, so I apologise to authority and to you.

